# Identification of Differentially Expressed Genes in Porcine Ovaries at Proestrus and Estrus Stages Using RNA-Seq Technique

**DOI:** 10.1155/2018/9150723

**Published:** 2018-02-14

**Authors:** Songbai Yang, Xiaolong Zhou, Yue Pei, Han Wang, Ke He, Ayong Zhao

**Affiliations:** College of Animal Science and Technology, Zhejiang A&F University, Lin'an, Zhejiang 311300, China

## Abstract

Estrus is an important factor for the fecundity of sows, and it is involved in ovulation and hormone secretion in ovaries. To better understand the molecular mechanisms of porcine estrus, the expression patterns of ovarian mRNA at proestrus and estrus stages were analyzed using RNA sequencing technology. A total of 2,167 differentially expressed genes (DEGs) were identified (*P* ≤ 0.05, |log_2_  Ratio| ≥ 1), of which 784 were upregulated and 1,383 were downregulated in the estrus compared with the proestrus group. Gene Ontology (GO) enrichment indicated that these DEGs were mainly involved in the cellular process, single-organism process, cell and cell part, and binding and metabolic process. In addition, a pathway analysis showed that these DEGs were significantly enriched in 33 Kyoto Encyclopedia of Genes and Genomes (KEGG) pathways, including cell adhesion molecules, ECM-receptor interaction, and cytokine-cytokine receptor interaction. Quantitative real-time reverse transcription polymerase chain reaction (qRT-PCR) confirmed the differential expression of 10 selected DEGs. Many of the novel candidate genes identified in this study will be valuable for understanding the molecular mechanisms of the sow estrous cycle.

## 1. Introduction

Sow fecundity is an important economic trait in the pig industry. The estrous cycle is a limiting factor for the fertility of sows, and it is involved in follicular development, ovulation, and hormone secretion in ovaries. Timely mating is the key to improving pregnancy rate and litter size. Therefore, the characterization of behavioral estrus, including swelling and reddening of the vulva, interest in the boar, and the standing reflex, is critical for mating [1]. In addition, the control of estrus and ovulation has become more important in recent years because of artificial insemination. The ovary is an important reproductive organ in mammalian animals and plays vital roles in follicle development and hormone secretion [2]. Follicle-stimulating hormone (FSH) and luteinizing hormone (LH) play essential roles in follicle maturation. During folliculogenesis, granulosa cells create the response to FSH and LH and then begin to produce oestradiol. As the ovarian follicle continues to grow and proliferate, the preovulatory stage begins [3, 4]. The pig estrous cycle spans 18–24 days, with the bulk of this time spent in the luteal phase (approximately 13–15 days). The follicular phase lasts 5–7 days. During this period, the selected antral follicles complete maturation with other follicles undergoing apoptotic or atresia [5–7].

Recently, the high-throughput RNA sequencing (RNA-Seq) technique has emerged as a useful tool for transcriptome analysis and exploring unknown genes [8]. Gene expression profiles during follicle development are complex. RNA-Seq has been applied to study ovarian follicle development of several livestock animals, such as goat [9, 10], sheep [11, 12], and cattle [13]. The use of the RNA-Seq technique identified many DEGs that were associated with pig fecundity [14–17]. A total of 11 genes identified in ovaries might be related to litter size in Yorkshire pigs [14]. Similarly, a large number of genes were downregulated in large litter size compared with the small litter size group in Berkshire pig placentas [16]. In the latest study, the transcriptome analysis of follicular tissue in diestrus and estrus from Large White and Chinese indigenous Mi gilts was also investigated, and a total of 2,838 DEGs were found in four different compared groups [17]. These studies have provided extensive insights into the understanding of significant genetic differences in pig fecundity. However, the basic molecular mechanism of the estrous cycle in sows, particularly in the period of proestrus and estrus stages, requires further study.

In the present study, to better understand the molecular factors and their regulatory genes involved in the estrous cycle, the mRNA expression profiles in ovaries of Landrace sows were compared between proestrus and estrus stages using the RNA-Seq technique. In total, 2,167 DEGs were identified. GO enrichment and KEGG pathway analyses showed that these DEGs were involved in cytokine-cytokine receptor interaction, cell adhesion molecules, and ECM-receptor interaction. These results provide novel insight into understanding the molecular mechanisms of the sow estrous cycle.

## 2. Materials and Methods

### 2.1. Ethics Statement and Experimental Animals

This study was reviewed and approved by the Animal Care and Use Committee of Zhejiang Agriculture and Forestry University (Lin'an, Zhejiang, China). Ovary samples were collected from three estrus and three proestrus Landrace multiparous sows. The six sows were 28 months old and they were at the fourth parity. The estrus sows were slaughtered at 24 h after exhibiting the standing reflex and the proestrus sows at 16 days after exhibiting the standing reflex. The corpora lutea were removed, and then the ovary samples were collected and frozen quickly in liquid nitrogen and then stored at −80°C. The ovary samples were homogenized for RNA isolation.

### 2.2. RNA Isolation, Library Construction, and Sequencing

Total RNA was isolated from six Landrace sows' ovaries in two groups using TRIzol reagent (Invitrogen, CA, USA), according to the manufacturer's instructions. The quality and concentration of RNA were determined by 1.2% agarose gels and the Agilent 2100 Bioanalyzer system (Agilent Technologies, CA, USA). Degradation of RNA was determined by 1.2% agarose gels. The concentration and purity of RNA were detected by the Nanodrop 2000 spectrophotometer (Thermo Scientific, MA, USA). Its integrity was confirmed using the Agilent Bioanalyzer 2100 system (Agilent Technologies, CA, USA). Sequencing libraries were generated using NEBNext1 Ultra™ RNA Library Prep Kit for Illumina (NEB, MA, USA). 3 *μ*g RNA per sample was used to purify mRNA using the oligo (dT) magnetic beads, and then the purified mRNA was randomly sheared into approximately 200 base pair pieces through the fragmentation buffer. The fragmented mRNAs were then used for first-strand cDNA synthesis by reverse transcriptase and random hexamer primers. Second-strand cDNA was synthesized using DNA polymerase I and RNase H. After the fragments were ligated to adaptors, the proper fragments through agarose gel electrophoresis were isolated as polymerase chain reaction (PCR) templates. The quality of the libraries was evaluated using an Agilent 2100 Bioanalyzer and the real-time PCR system. The libraries were sequenced using an Illumina HiSeqTM 2500 platform (Illumina, CA, USA).

### 2.3. Analysis of RNA-Seq Data

The sequences were removed according to the following criteria: low quality sequence (more than 30% of <Q20 bases) and more than 10% unknown nucleotides (N) reads and adapter. Then, the clean reads were acquired. The pig genome sequence (*Sus scrofa *10.2) [18] was downloaded from the ENSEMBL database (http://www.ensembl.org/index.html). All clean reads were mapped to the pig genome using HISAT software [19]. Transcripts assembly was performed by the Cufflinks software [20]. The gene expression level was calculated through the normalized number of fragments per kilobase of transcript per million fragments (FPKM) method [21]. Cuffdiff 2 software [22] was used to identify the DEGs between the estrus group and proestrus group using the following filter criteria: *P* value ≤ 0.05 and absolute value of log_2_ (FPKM_ESTRUS/FPKM_PROESTRUS) ≥ 1′′.

### 2.4. Gene Ontology and Pathway Enrichment Analysis

DEGs were annotated using the GO database (http://www.geneontology.org/) by hypergeometric test to examine the biological functions and pathways of these genes that were present in [23]. The *P* value was calculated, and GO terms were considered as significantly enriched when *P* value < 0.05. The background genes were genes involved in the whole genome. Pathway analyses were performed by the KEGG database (http://www.genome.jp/kegg/), and those with a *P* value < 0.05 were considered the significant pathways.

### 2.5. Quantitative Real-Time RT-PCR (qRT-PCR)

To confirm the transcriptome sequencing data, 10 candidate genes were selected and validated by qRT-PCR. 1 *μ*g of total RNA was used to synthesize cDNA using a reverse transcriptase kit (Takara, Dalian, China). The cDNA was used as the template for quantitative PCR by the SYBR Premix Ex Taq kit (Takara, Dalian, China). Quantitative PCR analyses were conducted using the CFX96 Touch real-time PCR system (Bio-Rad, CA, USA). The relative gene expression was normalized to RPL32 gene by the 2^−ΔΔCT^ method [24]. The PCR program was 95°C for 8 min and then 40 cycles of 94°C for 13 s and 60°C for 1 min; then the melting curve was run from 65–95°C with each amplification for three replicates. All primer sequences are listed in Table S1.

## 3. Results

### 3.1. Summary of Sequencing Data

In this study, 6 cDNA libraries from two groups (three from proestrus ovaries and three from estrus ovaries) were constructed and sequenced. The major characteristics of the sequencing and annotation data are described in Table 1. After low quality and adaptor sequences were filtered out, we obtained more than 38 million clean reads for six libraries. Among these clean reads, more than 96.54% and 91% had quality scores at the ratio of Q20 (a base quality > 20 and error rate < 0.01) and Q30 (a base quality > 30 and error rate < 0.001) level, respectively. There were 76.31%–80.94% of the clean reads mapped onto the pig reference genome (*Sus scrofa* 10.2). A total of 24,217 to 38,128 transcripts were obtained from the six libraries, and the average transcript length was approximately 2 kb.

### 3.2. Identification of DEGs

**Figure 1:**
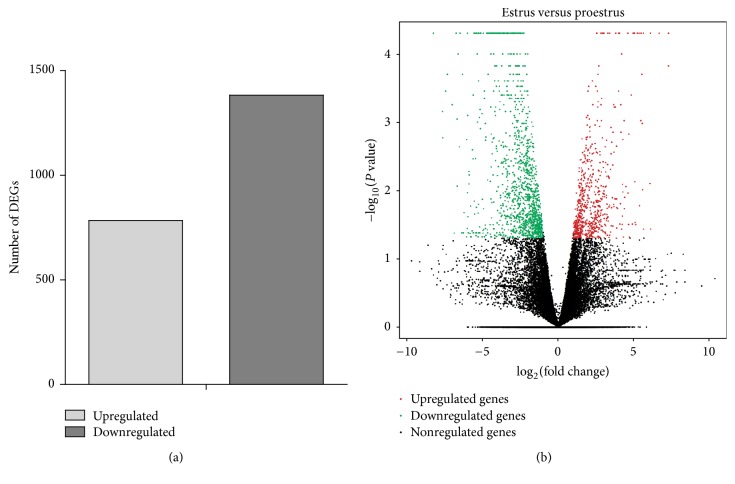
Distribution of DEGs. (a) The number of downregulated and upregulated DEGs in the estrus compared to the proestrus group. (b) Volcano plot displaying DEGs. The *y*-axis displays the value of −log10 (*P* value); the *x*-axis shows the log_2_ fold change value. The upregulated genes are displayed by the red dots; downregulated genes are displayed by the green dots; and the black dots represent genes with no significant changes.

A total of 30,369 genes were detected in the six cDNA libraries, and the FPKM method was utilized to evaluate the gene expression level. To analyze the transcriptome difference between proestrus and estrus stages, the estrus group was compared to the proestrus group. A total of 2,167 significant DEGs were identified, with 1,383 genes downregulated and 784 genes upregulated (*P* value ≤ 0.05 and |log_2_  FC| ≥ 1) (Figure 1 and Table S2).

### 3.3. Gene Ontology Enrichment Analysis

**Figure 2:**
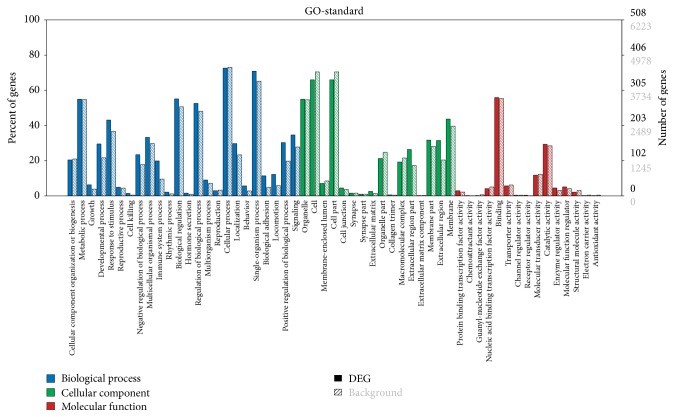
GO analysis of the DEGs. Genes were classified into biological process, cellular component, and molecular function. The left *y*-axis shows the percentage of genes in each category. The right *y*-axis indicates the number of genes in each category. The solid columns indicate DEGs, and slash columns indicate the background genes.

To further extend the molecular characterization of the DEGs, the DEGs were annotated using GO terms in the GO database. The DEGs were assigned to three categories, including biological processes, molecular functions, and cellular components (Figure 2 and Table S3). In the GO category biological process, DEGs were involved in the metabolic process, response to stimulus, biological regulation, cellular process, single-organism process, cell and cell part, binding and metabolic process, developmental process, cellular component organization or biogenesis, immune system process, and reproductive process. Among the DEGs related to the biological process, the most significant term was immune system process, containing 101 DEGs. Other enriched terms, including cell migration, cell chemotaxis, cell adhesion, and steroid biosynthetic process, were potentially associated with the estrous cycle. For cellular component annotation, there were 160 DEGs, with the most significant term located in the extracellular region (Table S3). The major molecular function category was binding (Figure 2).

### 3.4. Pathway Analysis

**Figure 3:**
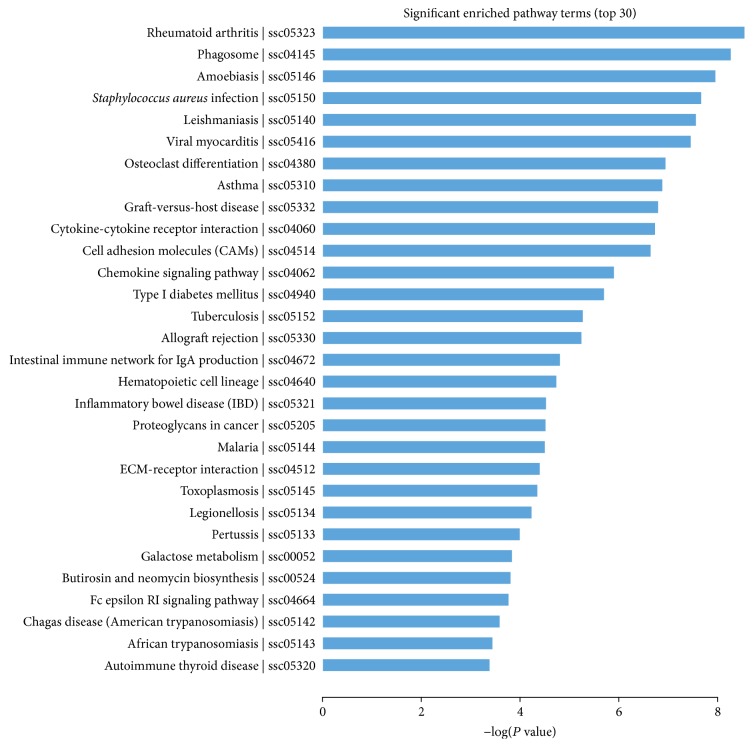
Top 30 significant enriched KEGG pathways.

A KEGG pathway analysis was performed to identify the pathways of the DEGs involved in the estrous cycle. In total, 1,700 DEGs were mapped to 239 KEGG pathways, and 32 pathways were significantly enriched (*P* ≤ 0.05) (Figure 3 and Table S4). In the significant pathways, several main pathways were represented, including cell adhesion molecules, cytokine-cytokine receptor interaction, and ECM-receptor interaction.

### 3.5. Validation of DEGs by qRT-PCR

**Figure 4:**
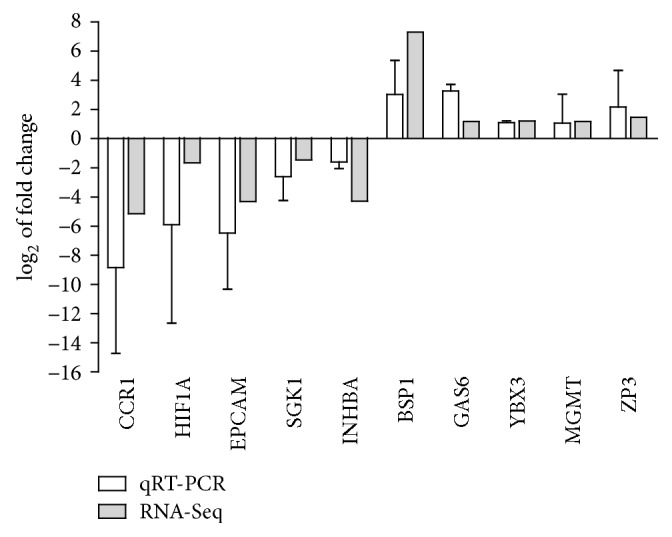
Validation of DEGs by qRT-PCR. White columns represent the expression level of the DEGs obtained by qRT-PCR, and gray columns represent the RNA-Seq results.

Ten candidate genes, including five downregulated genes, C-C chemokine receptor type 1 (CCR1), hypoxia-inducible factor 1-alpha (HIF1A), epithelial cell adhesion molecule (EPCAM), Inhibin beta A (INHBA), and serine/threonine-protein kinase Sgk1 (SGK1), and five upregulated genes, seminal plasma protein pB1 (BSP1), growth arrest-specific 6 (GAS6), Y box binding protein 3 (YBX3), O-6-methylguanine-DNA methyltransferase (MGMT), and zona pellucida sperm-binding protein 3 (ZP3), were selected and analyzed by qRT-PCR. Although the fold change varied between the two methods, trends in the expression of the 10 genes were consistent with the RNA-Seq results (Figure 4), indicating that the RNA-Seq results were reliable.

## 4. Discussion

The estrous cycle and estrus expression are crucial for the fecundity of sows. The estrous cycle involves the follicular development, ovulation, and hormone secretion in ovaries [7, 25, 26]. The RNA-Seq technique is a powerful approach for transcriptome analysis and exploring unknown genes [8]. Currently, the RNA-Seq technique has been performed in various reproductive systems, including ovaries [11, 12, 14], endometrium [27, 28], placenta [16, 29], testis [29], follicles [17], and granulosa cells [30] in poultry and livestock. In this study, RNA-Seq was utilized to identify the DEGs in ovary samples of proestrus and estrus pigs. A total of 2,167 genes were significantly differentially expressed in the estrus group versus the proestrus group, of which 784 were significantly upregulated and 1,383 were downregulated based on criteria of |log_2_  FC| ≥ 1 with *P* value ≤ 0.05. Ten DEGs were selected and verified by qRT-PCR analysis. GO and KEGG pathway analyses showed that these DEGs were involved in cellular process, single-organism process, cell and cell part, binding and metabolic process, cell adhesion molecules, ECM-receptor interaction, cytokine-cytokine receptor interaction, immune system process, reproductive process, cell migration, and steroid biosynthetic process. Further validations were performed by qRT-PCR for 10 selected DEGs, such as inhibin, beta A (INHBA), zona pellucida glycoprotein 3 (ZP3), and hypoxia-inducible factor 1-alpha (HIF1A). Previous research showed that INHBA inhibited FSH secretion and activity in granulosa cells and INHBA gene mutations were associated with litter size in sheep [31, 32]. In this study, the downregulated INHBA gene may contribute to an increase in FSH levels and facilitate follicular development in estrus porcine ovary. It has been reported that ZP3 functioned as the sperm receptor and mutations were associated with number of piglets born alive [32, 33]. In addition, HIF1A is required for vascular endothelial growth factor A (VEGFA) mediated ovarian follicle development and survival [34]. Thus, these genes may also play an important role in estrous cycle, and further research is required to investigate the function of these genes during proestrus and estrus stages.

The cellular process, single-organism process, binding, and metabolic process content are the basal process for granulosa cell growth and follicle development in proestrus and estrus stages. Our study showed that some DEGs were cytokine receptor related genes, such as IGF2R. IGF2R is downregulated in estrus versus proestrus, and the abundance of IGF-2 receptor (IGF2R) in granulosa cells (GCs) or theca cells is crucial for follicle growth and multiple ovulations [35, 36]. We also screened the gene IGFBP3 as a DEG. IGFBP3 is also important in follicle development [37]. Steroid hormones, including progestins, androgens, and estrogen, play important regulatory roles in the ovary by binding to their specific receptors and activating signal transduction pathways [38, 39]. The steroid biosynthetic pathway gives rise to progestins, androgens, and estrogen in the ovary and plays crucial roles in the reproductive process [38]. Our study showed that dozens of DEGs were hormone related genes and these genes were involved in steroid biosynthesis pathways. Eleven DEGs were classified into GO term steroid biosynthetic process (Table S3). Among these DEGs, the gene HSD17B1 encoding 17*β*-hydroxysteroid dehydrogenase 1 plays a vital role in estrogen metabolism and catalyzes the reversible reaction between estradiol and the less active estrogen, estrone [40]. One single nucleotide polymorphism (SNP) in intron 4 of the HSD17B1 gene was significantly associated with litter size, and these results showed that HSD17B1 could act as a potential molecular marker for litter size in pigs [41]. Another gene, CYP17A1, encoding the cytochrome p450c17a1 enzyme, regulates both steroid 17a-hydroxylase and 17,20-lyase activities, and it also plays a pivotal role in steroidogenesis [42]. HSD17B1 and CYP17 gene polymorphisms were associated with breast cancer risk; hence HSD17B1 and CYP17 represented possible drug targets for breast cancer treatment [43, 44]. CYP19A1 is also found as a DEG, and it is responsible for the aromatization of androgens into estrogen in follicles, affecting the granulosa cell proliferation and follicle growth in the proestrus stage [45]. In addition, SCAP gene was required for the full steroidogenic response through interaction with SREBP [46]. In steroid biosynthesis pathways, most of the DEGs were downregulated in the estrus group. A large number of genes were also downregulated in the estrus group compared with the diestrus group in Large White and Chinese indigenous Mi gilts follicles [17]. These results suggested that these DEGs were activated during the proestrus or diestrus stages. The function of these DEGs in the estrous cycle needs further investigation.

Moreover, GO categories of adhesion, including biological adhesion and cell adhesion, were classified into the top 10 GO categories (Table S3). The granulosa cells and oocyte of ovaries exist within a microenvironment, which does not come into direct contact with other cells [47]. An oocyte mainly interacts with its surrounding cells, including granulosa cells, through cell adhesion and connection [48]. We also found that the expression levels of genes related to cell adhesion molecules (CAMs) were significantly altered through the KEGG pathway analysis (Table S4). The CAMs pathway is consistent with the enrichment results in the adhesion GO category, further demonstrating that cell adhesion may play a major role in the estrous cycle of porcine ovary through different types of cell connections. Previous research showed that most of the DEGs were downregulated in the estrus stage compared with the diestrus stage in porcine follicle [17]. In this study, 22 DEGs were involved in the CAMs pathway, of which 20 DEGs were downregulated in the estrus stage. CAMs are proteins located on the cell surface that regulate the cell-cell or cell-substrate connections [49]. Previous research showed that CAMs play vital roles in embryonic implantation and ovarian follicle development [50, 51]. However, the function of these DEGs involved in CAMs in the estrous cycle should be further investigated. Furthermore, the results of the pathway analysis indicated that 12 genes, including DAG1, ITGA11, SDC1, CD44, ITGB3, ITGA3, FN1, and ITGA5, enriched in ECM-receptor interaction were downregulated. The ECM-receptor interaction pathways are involved in many biological processes, such as cell migration, proliferation, follicle growth, and oocyte maturation [52, 53]. Fifty-five genes enriched in the cell migration GO category were also identified, and most of these were downregulated in the estrus stage (Table S3). Therefore, we inferred that these genes might play vital roles in the transition from the proestrus stage to the estrus stage. Interestingly, the top two highest significant GO terms were immune system process and immune response, including 166 DEGs (Table S3). Many DEGs involved in immune response were also identified in estrus compared with the diestrus of porcine follicle [17]. However, the functions of these genes need to be further studied in the estrous cycle.

## 5. Conclusion

This study provides comprehensive transcriptome data on the porcine ovaries at proestrus and estrus stages through RNA-Seq technology. There were a total of 2,167 DEGs, of which 1,383 downregulated genes and 784 upregulated genes were identified. This study provides useful information for understanding the molecular mechanisms of sow estrous cycle. However, these transcriptome data are preliminary, and the function of the DEGs requires further investigation in estrous cycle.

## Figures and Tables

**Table 1:** RNA-Seq data statistics and annotation information results.

Samples	Estrus 1	Estrus 2	Estrus 3	Proestrus 1	Proestrus 2	Proestrus 3
Raw reads number	43,537,742	44,980,724	46,933,870	43,251,414	42,674,930	43,682,540
Raw bases	6,518,495,731	6,737,054,480	7,029,003,258	6,477,569,804	6,391,485,035	6,542,005,180
Clean reads number	39,553,500	40,781,504	42,823,178	38,910,588	38,772,168	39,721,342
Clean bases	5,921,488,722	6,107,904,887	6,413,011,132	5,827,088,794	5,806,734,458	5,948,483,660
Clean rate (%)	90.85	90.66	91.24	89.96	90.85	90.93
Q20 (%)	96.80	96.55	96.73	96.54	96.79	96.83
Q30 (%)	91.59	91.00	91.41	91.02	91.59	91.72
Mapped reads	30,184,209	32,430,870	34,341,651	31,166,703	31,287,495	32,149,872
Uniquely mapped reads	25,723,220	29,028,295	29,898,722	28,575,851	27,616,922	28,678,605
Multiple mapped reads	4,460,989	3,402,575	4,442,929	2,590,852	3,670,573	3,471,267
Transcript Number	24,217	38,128	28,636	37,748	31,381	30,867
Exon total length (bp)	36,281,115	83,700,310	49,116,462	89,558,585	64,040,781	57,382,487
Average transcript length (bp)	1,498	2,195	1,715	2,373	2,041	1,859
Max transcript length (bp)	8,370	19,865	11,037	22,330	32,100	13,205
Min transcript length (bp)	112	153	126	150	146	150
N50 length (bp, without intron)	1,986	3,061	2,326	3,361	2,800	2,525

## Supplementary Materials

Supplementary Materials Table S1: primer sequences for the genes selected for qRT-PCR. Table S2: DEGs identified under certain filter criteria. Table S3: GO enrichment analysis of DEGs. Table S4: KEGG pathway analysis of DEGs.
